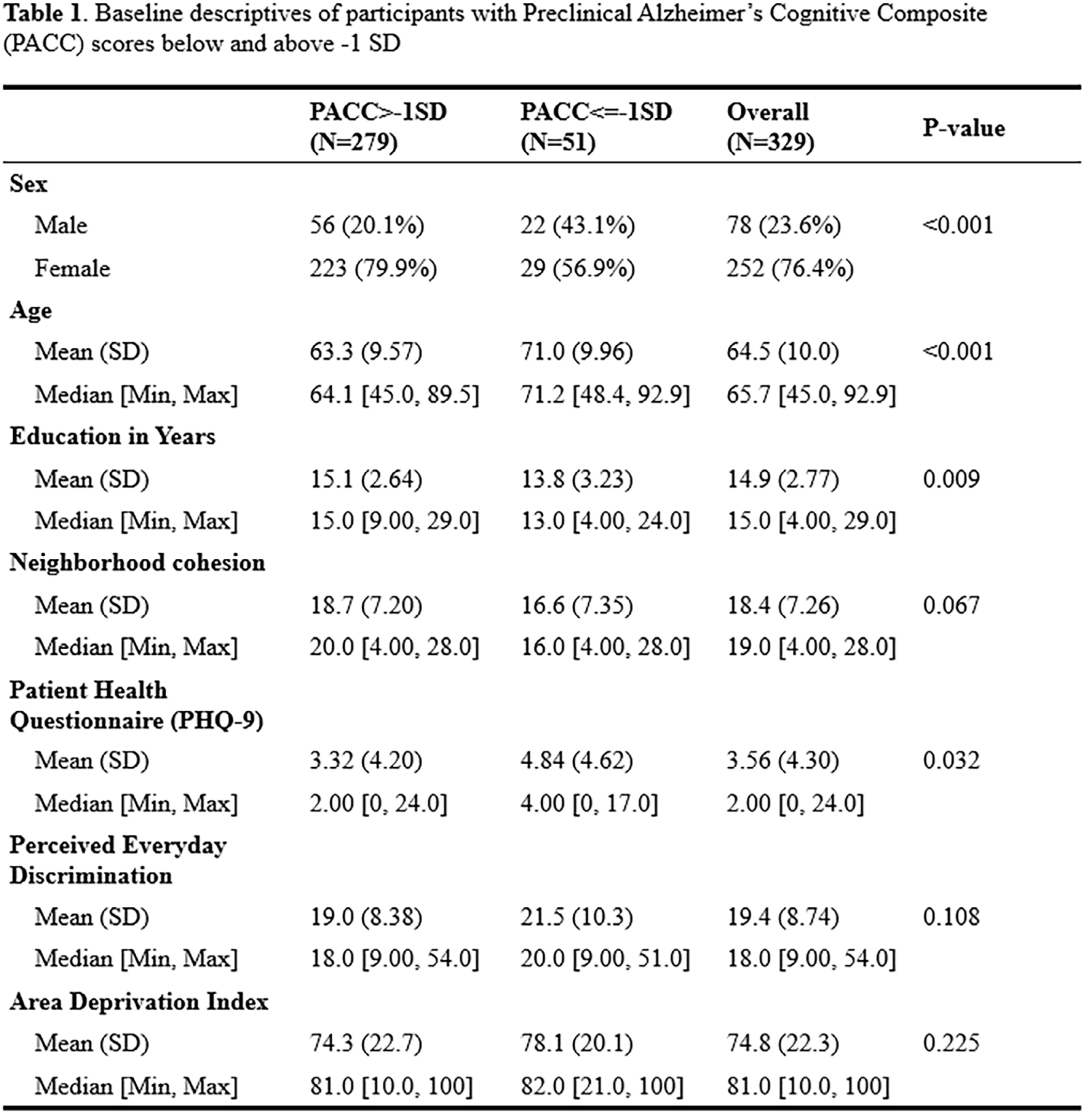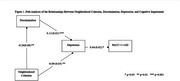# Community Matters: How Neighborhood Cohesion Buffers Against Cognitive Impairment via Mental Health Pathways in Black Older Adults

**DOI:** 10.1002/alz70861_108873

**Published:** 2025-12-23

**Authors:** Alexis I. B. Walker, Yiqi Zhu, Terri M. Buckner, Mario T. Millsap, Ramkrishna Kumar Singh, Semere Bekena, Ganesh M. Babulal

**Affiliations:** ^1^ Washington University School of Medicine, Saint Louis, MO USA; ^2^ Adelphi University, Garden City, NY USA; ^3^ Institute of Public Health, Washington University School of Medicine, Saint Louis, MO USA; ^4^ University of Johannesburg, Johannesburg, Gauteng Province South Africa

## Abstract

**Background:**

Little is known about how positive and sustained community socialization and support help to mitigate cognitive decline among adults racialized as Black, who are twice as likely to develop dementia. This study examined the associations between neighborhood cohesion and cognitive impairment, as well as the mediating effects of discrimination and depression.

**Method:**

We included 329 participants enrolled in the ARCHES Study who self‐identified as Black, 45 years of age or older, lived in the Greater St. Louis area, and completed a baseline visit. Cognition was assessed using a Preclinical Alzheimer Cognitive Composite score (PACC), including Trails A & B, Category Fluency‐Animals, and Free and Cued Selective Reminding Test. Neighborhood cohesion was measured using the eight‐item Neighborhood Disorder/Social Cohesion questionnaire, discrimination using the nine‐item Perceived Everyday Discrimination questionnaire, and depression using the Patient Health Questionnaire‐9. Structural equation modeling (SEM) was used to analyze the association between neighborhood cohesion and cognitive impairment, as well as the mediators, depression and discrimination, adjusting for age, sex, and education level.

**Result:**

The overall sample (*N* =329) was predominantly female (76.4%) with a mean age of 64.5 (range 45‐92) and an average of 14.9 years of education (Table 1). Participants with a PACC≤1 SD (*N* =51) were more likely to be male, be older, be less educated, report less cohesion, and report being more depressed. SEM revealed that depression mediated the association between neighborhood cohesion and cognitive impairment. A one‐point increase in neighborhood cohesion was associated with a ‐0.08 (SD=0.03, *p* <0.01) decrease in depression, and depression was associated with a 0.05 (SD=0.01, *p* <0.05)* increase in possible cognitive impairment (Figure 1). Discrimination was partially mediating the relationship between neighborhood cohesion and depression. A one‐point increase in neighborhood cohesion was associated with ‐0.21 (SD=0.06, *p* <0.01) decrease in discrimination, and a one‐point increase in discrimination was associated with 0.11 (SD=0.02, *p* <0.001) increase in depression. The area deprivation index was not associated with depression or cognitive impairment.

**Conclusion:**

Supportive mental health, social support, and positive interpersonal interactions with community members, regardless of neighborhood quality and socioeconomic disadvantages, are associated with better cognitive outcomes.